# Histological Type, Cytotoxic T Cells and Macrophages in the Tumor Microenvironment Affect the PD-L1 Status of Gastric Cancer

**DOI:** 10.3390/biomedicines11030709

**Published:** 2023-02-25

**Authors:** Tomislav Ivanović, Dorotea Božić, Benjamin Benzon, Vesna Čapkun, Katarina Vukojević, Merica Glavina Durdov

**Affiliations:** 1 Surgery Department, University Hospital in Split, Spinčićeva 1, 21000 Split, Croatia; 2 Department of Internal Medicine, University Hospital in Split, Spinciceva 1, 21000 Split, Croatia; 3 Department of Anatomy, Histology and Embryology, University of Split School of Medicine, Šoltanska 2, 21000 Split, Croatia; 4 Department of Pathology, Legal Medicine and Cytology, University Hospital in Split, 21000 Split, Croatia

**Keywords:** gastric cancer, tumor immune microenvironment, PD-L1, CTLA-4, IFN-γ

## Abstract

Gastric cancer (GC) therapies include gastrectomy and chemoradiotherapy. The tumor immune microenvironment (TME) has implications for potential immunotherapy. We analyzed the expression of PD-L1, CD8, CTLA-4 and IFN-γ in the tumor and regional lymph node (LN) of patients with GC and compared it with clinical and pathological data. Paraffin blocks were collected from 97 patients undergoing gastrectomy/lymphadenectomy for GC. Double immunohistochemistry was performed for CD8 and PD-L1 and double immunofluorescence for CTLA-4 and IFN-γ. Statistical significance was set at *p* < 0.05. PD-L1 expression in tumor cells was associated with intestinal GC type (*p* = 0.046), the density of macrophages and CD8 + T cells (*p* < 0.001, both). The median number of CD8+ T cells was higher in PD-L1-positive than in -negative tumors. A cut-off of 28.5 CD8 + T cells in one high-magnification field predicted PD-L1-positive tumors (AUROC 0.797, sensitivity 74.2%, specificity 77.3%). IFN-γ expression in tumor cells was found in 37 GCs and was positively associated with CTLA4^+^ lymphocytes in the LN (*p* = 0.027) and CTLA4^+^/IFN-γ+ in tumors and the LN (all *p* < 0.001). The median overall survival (OS) was 17 months. In the group of deceased patients, IFN-γ expression in metastases correlated with lower OS (RHO = −0.314, *p* = 0.008). PD-L1 expression in tumor cells correlated with CD8 + T cells and macrophages in the TME and IFN-γ expression with suppressive CTLA4^+^/IFNγ+ immune cells in the TME and LN.

## 1. Introduction

Gastric cancer (GC) is the fifth most common malignancy and the third leading cause of cancer-related death [1]. The incidence of GC in Central and Eastern Europe has been estimated at 17.1 per 100,000 men and 7.5 per 100,000 women. GC usually occurs in patients over 50 years of age and is 2–3 times more prevalent in men [2]. Chronic H. pylori infection is a group I carcinogen for GC, followed by EBV infection, autoimmune and atrophic gastritis, Menetrier’s disease and radiation exposure [3]. A diet with a high salt content, smoking tobacco, drinking alcohol, consumption of red and processed meat and a low level of physical activity can contribute to the development of GC [4]. Proper preventive measures include H. pylori screening and eradication and an upper endoscopy screening program for early cancer detection [5]. The definitive therapy is gastrectomy and chemotherapy. However, recently, immune checkpoint inhibitors have emerged as a promising therapeutic modality [6].

A better understanding of the molecular mechanisms of the immune tumor microenvironment (TME) in GC is becoming an imperative focus of a new therapeutic paradigm. The TME comprises cells in the extracellular matrix surrounding tumor cells, including endothelial cells, cancer-associated fibroblasts, macrophages and dendritic cells, B cells and T cells [7]. The TME is a suitable living environment for cancer cells and cancer stem cells, ensuring their growth, immune evasion and resistance to therapy [8,9]. The presence and density of tumor-associated macrophages are correlated with poor prognosis and resistance to GC treatment [10]. Antigen-specific cytotoxic CD8^+^ T cells directly kill tumor cells or mediate their apoptosis through the Fas/FasL signaling pathway [11]. After the initial antigen stimulation is removed, the memory cells shrink and are found in the tissue and lymph nodes.

In a mouse model, TME-resident memory cells significantly enhance antitumor capacity and secrete interferon-γ (IFN-γ) to inhibit tumor progression [12]. A low level of these cells (CD8^+^ CD103^+^) is associated with a worse prognosis. Activated T cells in the TME experience prolonged antigen exposure/mediation of APC and cytokines, and they express inhibitory molecules to limit tissue damage [13]. These CD8^+^ T cells have consistently high expression of immune checkpoint molecules such as PD-1 (programmed cell death-1), TIM-3 (T-cell immunoglobulin and mucin-domain-containing-3) and CTLA-4 (cytotoxic T-lymphocyte antigen-4) [14]. IFN-γ expressed by lymphocytes stimulates an immune reaction, but IFN-γ co-expressed with CTLA-4 labels deeply suppressed T cells [15]. PD-L1 on macrophages and tumor cells is regulated by inflammatory signaling, oncogenic signaling and genetic and epigenetic alterations [16]. Immune checkpoint inhibitors such as anti-PD-L1 (atezolizumab), anti-PD-1 (nivolumab and pembrolizumab) and anti-CTLA-4 (ipilimumab) are currently not widely used in the treatment of GC [17]. This study aimed to analyze the clinical and pathological features of GC in patients who underwent gastrectomy and compare them with the expression of PD-L1, CTLA-4, CD8+ and IFN-γ in the TME, in order to evaluate their relationship and possible influence on survival.

## 2. Materials and Methods

### 2.1. Patient Cohort

The sample contained 97 patients who underwent gastrectomy due to GC from 2014 to 2018 in the University Hospital of Split, Croatia. Histological slides and paraffin blocks of tumor tissue were collected from The Department of Pathology. In addition, data on age, sex, date of diagnosis and pathological stage according to the eighth edition of the *AJCC* Staging Manual, Lauren pathohistological classification (intestinal, diffuse, mixed), Borrmann type of advanced cancer (polypoid, fungal, ulcerative, flat/infiltrative) and disease stage (IA-IV) were collected from the electronic medical records. New molecular classification according to The Cancer Genome Atlas (EBV-associated, MSI-associated, with chromosomal instability and genomic stability) was not applied because it was not routinely tested by molecular methods [18]. Patients with complete clinical data and paraffin blocks of tumor tissue were included in the study. Patients who had gastroesophageal junction tumor, synchronous cancer, incomplete clinical data or received preoperative chemo/radiotherapy were excluded.

### 2.2. Immunohistochemistry

**Figure 1:**
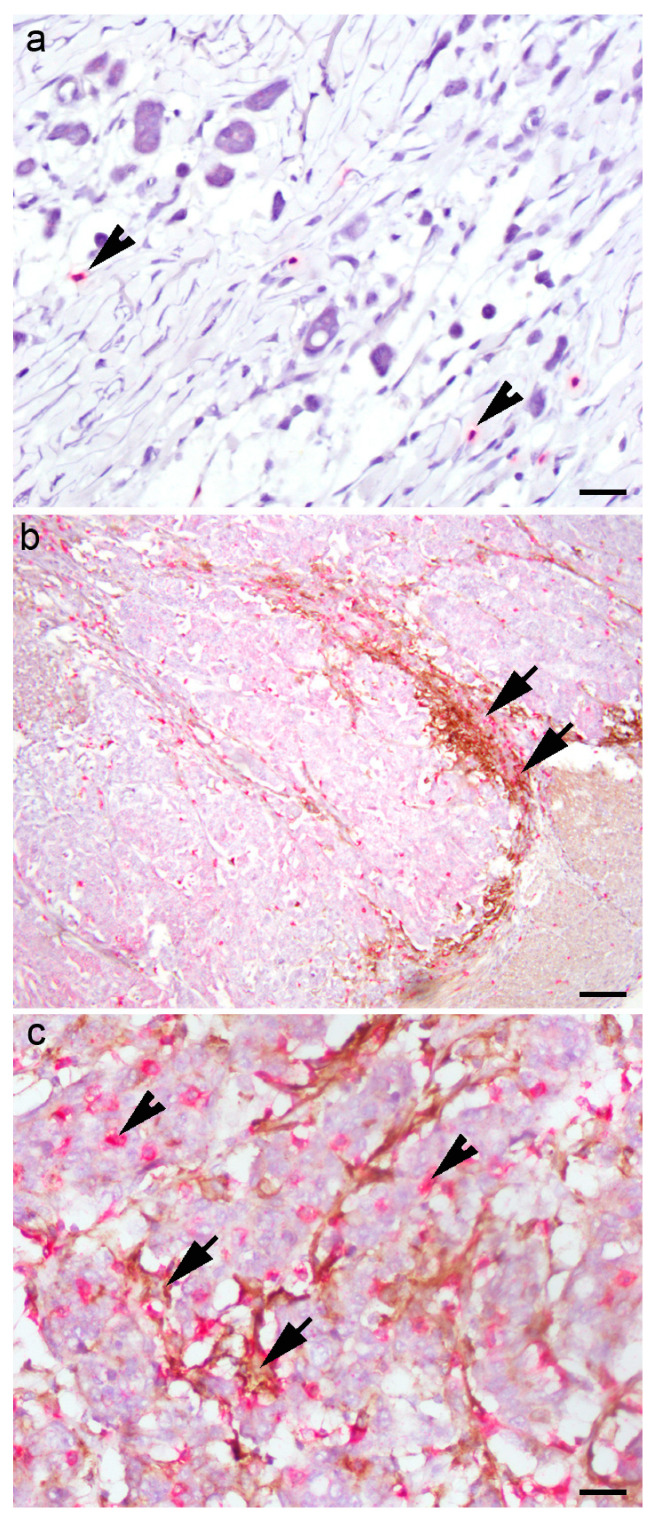
Double immunohistochemistry of PD-L1 and CD8 in tumor microenvironment of gastric cancer: (**a**) “desert landscape” with few CD8+ T immune cells (arrowheads) and PD-LI-negative tumor cells; (**b**) PD-L1-positive macrophages (arrows) in a broad front against PD-L1-negative tumor cells; (**c**) PD-L1-positive tumor cells intermingled with PD-L1-positive macrophages (arrow) and CD8+ lymphocytes (arrowheads). Scale bar (**a**,**c**) 50 µM, (**b**) 80 µM.

Five-micrometer slides were cut from the paraffin blocks, deparaffinized, rehydrated and stained on Ultra Benchmark (Ventana Medical Systems, Inc. Oro Valley, AZ, USA). Double immunohistochemistry was performed using rabbit monoclonal anti-CD8 (SP57, Ventana) and monoclonal mouse anti-PD-L1 (SP263, Ventana) and appropriate secondary antibodies (DAB/ICH and Fast red/ICH Ventana, Basel, Switzerland). Positive CD8 expression was determined by red cytoplasmic staining, and positive PD-L1 expression was established by brown membrane staining. The external controls for CD8 and PD-L1 were tonsillar and placental tissue, respectively. The negative control was the slides processed without the application of primary antibodies. The slides were analyzed on an Olympus 41 BX light microscope (Olympus, Tokyo, Japan) by two experienced pathologists. PD-L1-positive tumor cells and PD-L1-positive macrophages differed morphologically (inset on Figure 1). The density of macrophage infiltration was valued as 0 (no infiltration), 1 (sparse), 2 (peripheral) and 3 (dense and intermingled with tumor cells), as we described previously (Figure 1) [19].

The presence of PD-L1-positive tumor cells was analyzed on four randomly chosen HPMF and estimated as negative (0), low (1–10%), moderate (11–49%) and strong (≥50%). The PD-L1 status of GC was considered positive if ≥1% tumor cells expressed PD-L1. The absolute number of CD8^+^ T cells on 4 HPMFs was analyzed, and the mean in 1 HPMF was counted for each patient.

**Figure 2:**
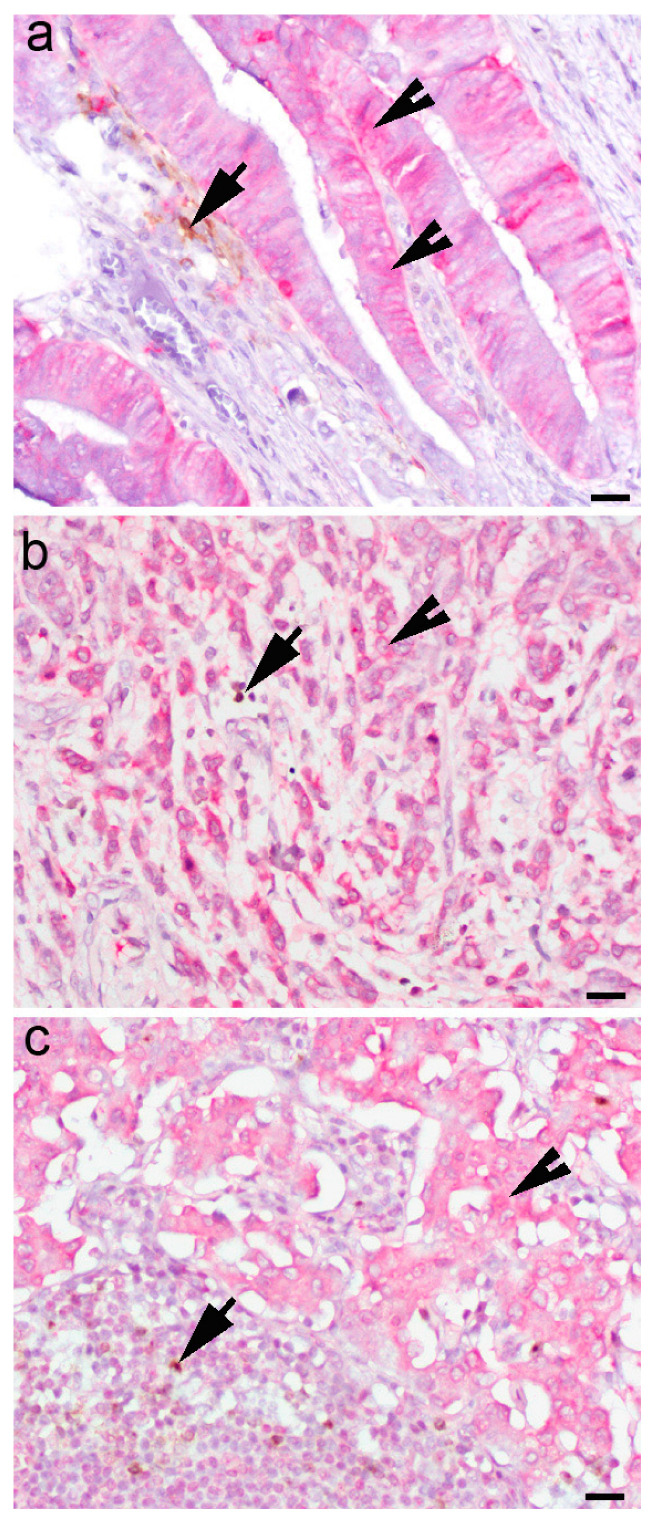
IFN-γ expression in the cytoplasm of (**a**) dysplastic epithelial cells in gastric mucosa and (**b**) a diffuse type of gastric cancer and tumor cells in metastatic lymph node. (**c**) IFN-γ-positive cells (arrowheads), CTLA4^+^ cells (arrows), indirect double immunohistochemistry, chromogen DAB for CTLA4 and fast red for IFN-γ. Scale bar (**a**) 50 µM, (**b**) and (**c**) 80 µM.

Immunohistochemistry for IFN-γ was performed to confirm IFN-γ expression in tumor cells and the TME (Figure 2).

### 2.3. Immunofluorescence

The slides underwent the same procedure as for immunohistochemistry. To avoid nonspecific staining, a protein-blocking buffer (ab64226, Abcam, Cambridge, UK) was administered for 30 min. The samples were then incubated with primary antibodies overnight in a humidity chamber. They were incubated the following day, with suitable secondary antibodies for one hour. Indirect double immunofluorescence with monoclonal rabbit CTLA-4 (233772 Abcam, Cambridge, UK) and monoclonal mouse IFN-γ (218426, Abcam, Cambridge, UK) was performed manually, using secondary antibodies donkey anti-mouse Alexa flour and donkey anti-rabbit Rhodamine Red, which is a standard procedure. The nuclei were stained with 40,6-diamidino-2-phenylindole (DAPI) (Immuno-Mount, Thermo Shandon, Pittsburgh, PA, USA).

**Scheme 1:**
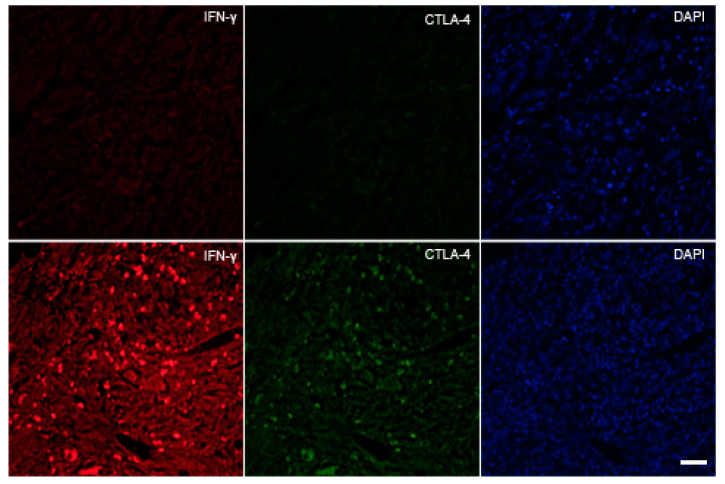
First-row negative control, second-row positive control (lymph node). Scale bar 25 µM.

The omission of the primary antibody from the immunofluorescence protocol led to virtually no staining on the slides, implying that the secondary antibody did not cross-react with the tissue section (secondary antibody control). For primary antibody control, they were diluted separately in the same concentrations as for the staining protocol but with a blocking solution and each corresponding peptide antigen. The sections were then incubated with appropriate mixtures. Again, no staining of the slides was observed. Additionally, neither nonspecific secondary antibody binding nor false-positive results were seen (Scheme 1).

### 2.4. Data Processing

The slides were analyzed on an immunofluorescence microscope, Olympus X41, on high magnification (400×), and 15 representative slides were photographed (5 from the tumor infiltration area (TI), 5 from the invasive tumor margin (IM) and 5 from the lymph node (LN) with a digital camera, ORCA-spark, Hamamatsu, Japan. The microphotographs were merged with the cellSens Dimension software program (Olympus, Tokyo, Japan) and analyzed manually on the screen. The IFN-γ signal was presented as a red signal, CTLA4 as a green signal, and the yellow signal was interpreted as a co-expression of CTLA4 and INF-γ in one immune cell. The absolute number of CTLA+ cells and CTLA/IFN+ cells was counted manually.

The last day of clinical follow-up was December 31, 2019. Duration of follow-up was 72 months, mean of 32 months, median of 17.5 months. OS was calculated from the date of diagnosis to I) the date of death from any cause, or II) determined as alive/deceased on the last day of follow-up. All data were organized in Excel sheets and statistically analyzed. The study was conducted according to the Declaration of Helsinki and was approved by the ethics committee of the University Hospital in Split (number 500-03/18-01/18).

### 2.5. Statistical Analysis

The Statistical Package for the Social Sciences (SPSS) software (version 19 for Windows; SPSS Inc., Chicago, IL, USA) was used in the statistical data processing. Categorical variables were analyzed as count and frequency, and continuous variables as mean, SE and 95% CI. For normally distributed continuous variables, a t-test was applied, and for abnormally distributed continuous variables, the nonparametric Mann–Whitney U test was used. For the comparison of categorized variables, the χ^2^ test was used. Correlation was assessed using the Pearson correlation coefficient. The Kaplan–Meier curve and log-rank test were applied for analyses of overall survival. The significance level was *p* < 0.05.

## 3. Results

### 3.1. Demographic and Clinicopathological Characteristics

This retrograde cross-sectional study included 97 patients with GS who underwent gastrectomy from 2014 to 2018 in the University Hospital of Split, Croatia. Demographic and clinicopathologic characteristics are shown in Table 1. There were 66 (68%) men and 31 (32%) women. The average age was 72 years (Q1–Q3: 65–80; min–max: 46–87), with no significant difference between genders (Z = 0.170; *p* = 0865).

### 3.2. Correlation between PD-L1 Status of Gastric Cancer and Clinicopathological Parameters

A total of 31/91 (31.9%) patients had a PD-L1-positive GC status. The correlation of the PD-L1 GC status with clinical and pathological parameters is shown in Table 2

**Figure 3:**
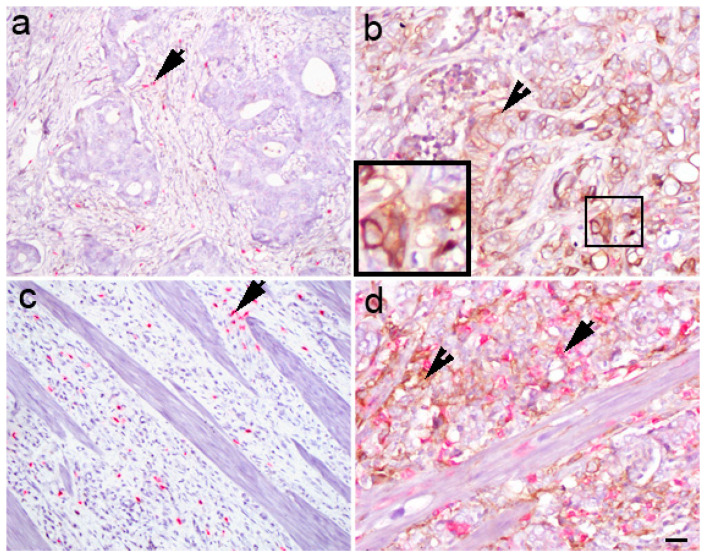
Double immunohistochemistry with PD-L1/CD8 staining in gastric cancer. Different PD-L1 expression levels (arrowheads) in gastric cancer: negative (stage 3B) (**a**) and positive (stage 2B) (**b**) in intestinal type, and negative (stage 4B) (**c**) and positive (stage 3B) (**d**) in diffuse type. CD8^+^ cells are red (arrows). Scale bar 25 µM.

PD-L1 was associated with the histological type (χ^2^ = 3.97; *p* = 0.046). The proportion of diffuse type was 2.2 times higher in the PD-L1-negative group than in the PD-L1-positive cases. The diffuse type was 3.1 times more common in PD-LI-negative than PD-L1-positive GC (OR = 3.1; 95%CI: 1.18, 5; *p* = 0.030) (Figure 3).

The proportion of diffuse type was 2.2 times higher in the PD-L1-negative group than in the PD-L1-positive cases. The diffuse type was 3.1 times more common in PD-LI-negative than PD-L1-positive GC (OR = 3.1; 95%CI: 1.18, 5; *p* = 0.030). Positive expression of PD-L1 in tumor cells was associated with higher infiltration of macrophages (χ^2^ = 32.8; *p* < 0.001). Moderate and dense macrophage infiltration were 7.2 and 4.8 times more frequent in PD-L1-positive tumors than in PD-L1-negative tumors. Mild infiltration of macrophages was five times more common in PD-L1-negative tumors. For each increase in the density of macrophage infiltration, the probability of a PD-L1-positive (1, 2, 3) compared to a PD-L1-negative (0) finding increased 3.6 times (OR = 3.6; 95%CI =2.1–6), 1; *p* <0.001). The prevalence of negative macrophage infiltration in PD-L1-negative tumors was five times higher than the prevalence of macrophages (1, 2, 3) combined. The ratio of macrophages (1, 2, 3) in the PD-L1-positive GC was 2.6 times higher than that of macrophages (1, 2, 3) in the PD-L1-negative cases (χ^2^ = 22.3, *p* < 0.001). The probability of the appearance of macrophages (1, 2, 3) compared to macrophages (0) was 13.5 times higher in PD-L1 (1, 2, 3) tumors compared to PD-L1 (0) cases (OR = 13, 5; 95%Cl: 4.2–43; *p* < 0.001).

**Figure 4:**
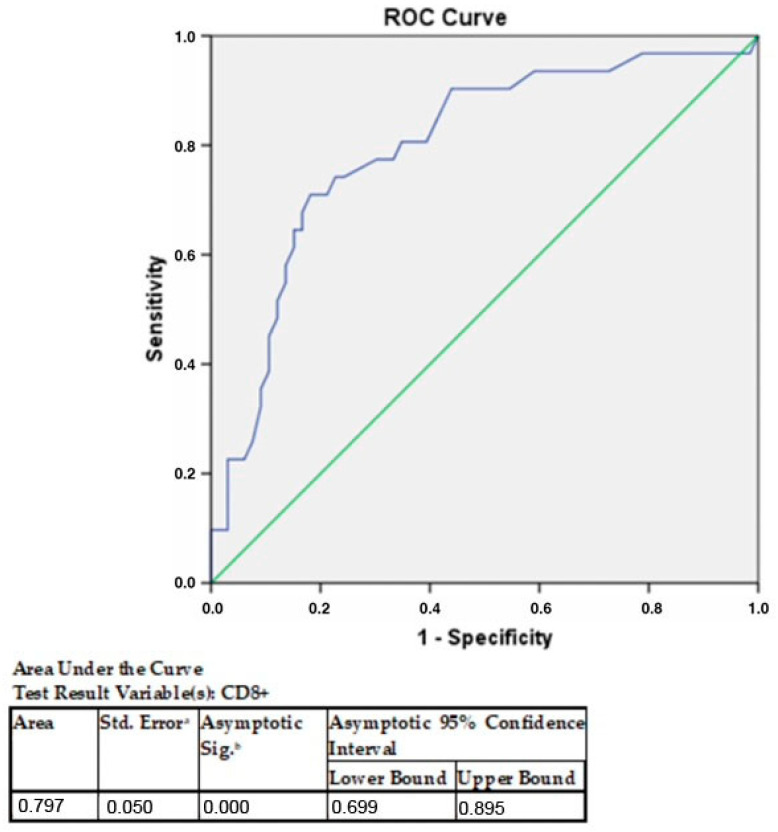
AUROC analysis of CD8^+^ cells in GC according to PD-L1 expression in tumor cells. CD8^+^ T cells have at least one tie between the positive actual state group and the negative actual state group. The statistics may be biased ^a^ under the nonparametric assumption. ^b^ Null hypothesis: true area = 0.5.

PD-L1 and CD8^+^ T cells were significantly correlated (χ^2^ = 21.3; *p* < 0.001). Again, groups were formed with ≥28.5 CD8^+^ T cells and <28.5 CD8^+^ T cells. The proportion of cases with <28.5 CD8^+^ T cells was three times higher in PD-L1-negative GC than in positive ones (Figure 4).

The proportion of cases with ≥28.5 CD 8^+^ T cells was three times higher in PD-L1-positive GC than in negative cases (χ^2^ = 21.3; *p* < 0.001). The probability of PD-L1-positive compared to PD-L1-negative GC in cases with ≥28.5 CD8^+^ T cells was 9.8 times higher than in cases with <28.5 CD8^+^ T cells. No association was found between PD-L1 and disease stages (IA, IB; IIA, IIB; IIIA, IIIB, IVB). The proportion of stages IA and IB was 1.7 times higher in PD-L1-negative cases than in PD-L1-positive cases; stage IIA and IIB in PD-L1-positive cases were two times more common than in PD-L1-negative cases.

CTLA4^+^ lymphocytes did not correlate to PD-L1 expression in the TI or IM (χ^2^ = 1.7; *p* = 0.188, and χ^2^ = 1.1; *p*= 0.301, consecutively), nor in the lymph node (χ^2^= 0.833; *p* = 0.361). Furthermore, other analyzed parameters did not significantly correlate with the PD-L1 status of tumor.

Multivariate logistic regression was performed for PD-L1 concerning histologic type, macrophage infiltration, CD8^+^ T cells and TNM stage (Table 3).

The association between macrophages and CD8^+^T cells with PD-L1 was confirmed (*p* < 0.001, both). Macrophages were 9.8 times more prevalent in PD-L1-positive than in PD-L1-negative tumors (*p* < 0.001).

### 3.3. Correlation of IFN-γ Expression in Gastric Cancer with Clinicopathological Parameters

The expression of IFN-γ in tumor cells was found in 37 cases, which were further analyzed as IFN-γ-positive. IFN-γ expression in tumor cells correlated to the presence of CTLA4^+^ lymphocytes in lymph nodes (Z = 2.2; *p*= 0.027) (Table 4). The absolute number of CTLA4^+^ cells in lymph nodes was 1.6 times higher in IFN-γ-positive than in IFN-γ-negative tumors. Patients with IFN-γ-positive tumors had a 1.6 greater prevalence of CTLA4^+^ cells in lymph nodes than those with IFN-γ-negative tumors. On the other hand, the cases without CTLA4^+^ cells in lymph nodes were 1.5 times more common in the IFN-γ-negative tumor group (χ2 = 3.6; *p* = 0.059). The odds ratio for the presence of CTLA-4 concerning the non-presence of CTLA-4 was 2.4 times higher in IFN-positive tumors than in IFN-negative tumors (OR: 2.4; 95% CI: 1.1–5.6; *p* = 0.037).

The incidence of lymph nodes with CTLA4^+^ lymphocytes compared to those without CTLA4^+^ was 2.4 times higher in IFNγ-positive tumors than in IFNγ-negative tumors (OR: 2.4; 95% CI: 1.1–5.6; *p* = 0.037).

**Figure 5:**
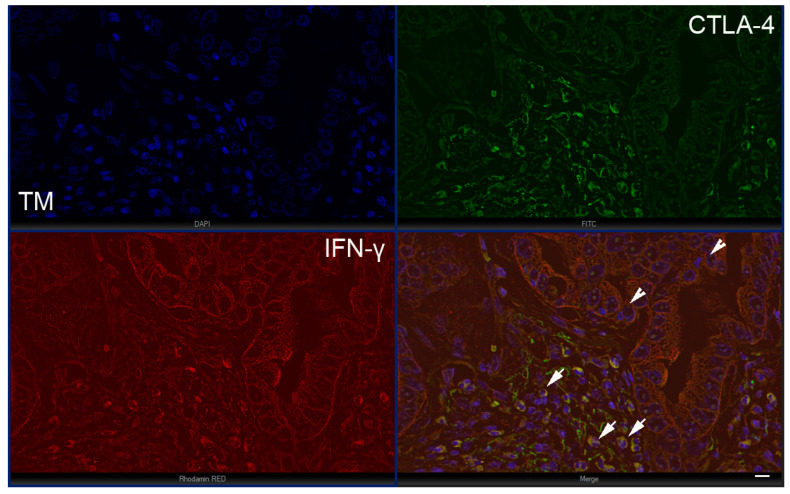
The expression of IFN-γ and immunosuppressive T cells in the tumor microenvironment at tumor margin (TM); intestinal type (stage 1A) with IFN-γ-positive tumor cells (arrowheads) and a significant number of CTLA-4^+^/IFN-γ^+^ T lymphocytes (arrows). Scale bar 25 µM.

CTLA4^+^/IFN-γ^+^ lymphocytes in the lymph node correlated with PD-L1 in the tumor at a significance level of 93% (χ^2^ = 7.1; *p*= 0.069) (Table 5) (Figure 5).

Out of 60 cases with negative IFN-γ expression in tumor cells, all had negative IFN-γ expression in TME lymphocytes and lymph nodes (Table 6).

Quite the opposite, all 37 cases with IFN-γ-positive tumor cells had INF-γ-positive lymphocytes in the TI and TM, as well as in lymph nodes (*p* < 0.001 for all).

The mean OS was 32 months (SE: 3.2; 95% CI: 25.5–38), and the median OS was 17.5 months (SE: 3.9; 95% CI: 9.8–25) (Table 7).

### 3.4. Analysis of Overall Survival of Patients Who Underwent Gastrectomy Due to GC

The mean OS was 27.5 months longer in stages IA and IB than in IIA and IIB and 30 months longer than in IIIA, IIIB and IVB. Therefore, the probabilities of 3-year survival for stages IA and IB, IIA and IIB and IIIA, III B and IVB are 71.4% (SE = 12%), 32.3% (SE = 8.4%) and 23% (SE = 6%), respectively (Table 7).

By the end of follow-up, 71 (73%) patients had died. The median age of the deceased was 76.6 years (Q1-Q3: 66–85; min–max: 46–87). Spearman’s correlation coefficient of survival for decreased patients with IFN-γ-positive tumor cells was -0.162 (*p* = 0.176). A negative correlation was found between deceased patients and positive IFN-γ expression in metastatic tumor cells, RHO = −0.314 (*p* = 0.008). The risk of death increased 1.6 times with each increase in the stage (IA, IB; IIA, IIB; IIIA, IIIB, IVB) compared to the previous one. When all stages, IA-IVB, were included, Cox’s regression analysis showed that the risk of death increased by 1.4 times with each increase in the disease stage compared to the previous one (HR1.4; 95% CI 1.2–1.6; *p* < 0.001).

## 4. Discussion

Histological types of GC differ in pathogenesis and etiology. Such a background is reflected in the TME, a complex biological system closely related to cancer development, progression, metastasis and immune evasion [8]. Mashukov et al. defined three types of immune cell infiltration: an immune desert, immune excluded and inflamed pattern [20]. According to the expression of PD-L1 and CD8^+^ T cells, Koh et al. categorized four types of immunological microenvironments [21]. Immunophenotype influences PD-L1 expression. In our study, expression of PD-L1 was found in 31.9% of tumors, lower than in a Chinese study, 42.2%, and higher than in a Korean study, 25% and in a Ukrainian study, 18% [20,21,22]. Li et al. divided GC into two groups according to low and dense lymphocyte infiltration [23]. The authors found that massive lymphocyte infiltration and the diffuse/mixed type of histology were independent factors for PD-L1-positive GC status. In an immunohistochemical study of the TME in GC, Mashukov et al. found higher PD-L1 expression in the intestinal type with an inflammatory pattern than in the diffuse and mucinous type [20]. They calculated the positive expression in tumor cells and macrophages as a combined positive score. In our study, the intestinal type of GC was more commonly associated with PD-L1 expression than the diffuse type. CD8^+^ T-cell density was positively associated with the PD-L1 status of GC, with a median of 27 cells in a one HPVP. Mashukov et al. also found an association between PD-L1 status and CD8^+^ T cells, counting 84 lymphocytes in 1 mm^2^ as a median, to separate low- and high-density CD8^+^ cells in the TME of GC [20]. Wang et al. estimated a median of 24 CD8^+^ T cells per HPMF to classify high/low cytotoxic cell infiltration [24].

Koh et al. found that MSI-high and EBV+ GC were most common in the high-PD-L1^+^CD8-type group [24]. The same results described by De Rosa E et al. highlight this type as the most promising target for GC immunotherapy [25]. Wang et al. also concluded that EBV+ GC patients have a shorter survival than EBV patients [24]. According to Kim et al., the presence of a pre-existing immune response to GC varies according to the pathobiology of the tumor molecular subtype, which determines the effectiveness of immunotherapy [26]. Intestinal type, a high density of CD8+ lymphocytes and CD68^+^ macrophages, and EBV-associated and MSI-associated GS are connected with higher PD-L1 expression. In our study, MSS was not analyzed, but one patient with EBV+ GS, as confirmed by EBER in situ hybridization, had an inflamed TME with numerous CD8^+-^T lymphocytes and a positive PD-L1 status.

Tumor-associated macrophages, which secrete the factors included in tumor progression, are the most abundant immune cells in the TME. We found a correlation of macrophages with PD-L1 expression in tumor cells (*p* < 0.001) and a progressive trend towards each grade of macrophage density (*p* < 0.001). Mashukov et al. found that the number of M2 macrophages within tumor clusters and peritumoral areas was the highest in the intestinal type of GC, and the number of M1 and M2 macrophages was highest in the inflammatory immunophenotype [20].

In our sample, the mean OS was 17 months, and PD-L1 expression and CD8^+^ T cells did not affect OS. In a GC study from 2006, PD-L1 expression was associated with tumor aggressiveness and unfavorable outcome [23]. Shigemori et al. found soluble PD-L1 in the circulation to be a predictive marker for gastric cancer recurrence [27]. According to a study by Koh et al., PD-L1 alone had no impact on OS, but in the context of CD8^+^ T cells, patients with the PD-L1^low^ CD^high^ immune type had the best OS, and PD-L1^low^ CD8^low^ the worst OS [20]. Oki et al. also found that PD-L1 was associated with a worse prognosis in surgically treated GC patients [28]. They concluded that the co-localization of an inflammatory response with PD-L1 expression in tumors indicates an adaptive resistance mechanism of immune escape. In this context, CD8^+^ T cells are an active immune factor, PD-L1 expression is an immune inhibitory factor, and the TME equalizes the prognostic window based on immunological classification. Although it does not affect the chemotherapy outcome, immune classification allows for the selection of those who respond to PD-L1 blockade.

The patients most at risk (high CD8 T-cell prevalence simultaneously accompanied by high PD-L1 expression) are those with worse OS, who need improved treatment options. In our study, PD-L1 expression was associated with the density of CD8^+^ T cells in the TME. According to the literature, PD-L1-positive cases have a worse prognosis than PD-L1-negative cases [22,23]. Wu et al. emphasize the association of PD-L1 with tumor size, invasion, lymph node metastasis and shorter survival [22]. Thompson et al. found that PD-L1-positive cancer/stromal cells, and a high density of CD8^+^ T cells, correlated to lower DFP and OS in all GC stages [29]. In a study by Wang et al., CD8 T-cell density was associated with the EBV status of gastric cancer and was found to lead to a worse outcome than EBV-negative cases [24]. The authors evaluated the immune response of tumor-infiltrating CD8^+^ T cells and the expression of PD-L1 in the area of tumor infiltration. They found that patients with a weak immune reaction had a shorter survival than patients with a strong immune response. Finally, they recommended a new immune classification of GC for better prediction of prognosis and selection for immunotherapy. The response rate of immunotherapy drugs targeting PD-L1, PD-1 and CTLA-4 is 20–40%. Identifying biomarkers to better select responders is an important goal [30]. Mao et al., in a meta-analysis of 22 studies, found that the pre-treatment peripheral cytokine levels of IL-6 and IL-8, but not IFN-γ, are predictors of worse OS of cancer patients treated with immune checkpoint inhibitors [31]. Additionally, one potential therapy is represented by the production of an anti-PDL1 vaccine that blocks PD-1/PD-L1 interaction, as a promising new therapeutical approach [32]. Sancez-Zaucho et al. proposed IL-6, IL-10 and IFN-γ as potential diagnostic markers in GC because of their significantly higher circulating levels in GC patients than in healthy control [33]. Together with IL-10, IFN-γ might be helpful for diagnosing early-stage GC. However, the role of IFN-γ in GC promotion is controversial because it is proinflammatory upregulated in the gastric mucosa after chronic Helicobacter infection, and on the other hand, has immunosuppressed activities.

The expression of IFN-γ in gastric mucosa and tumor cells was an unexpected finding in this investigation. IFN-γ expression was confirmed in tumor epithelial cells via immunofluorescence and immunohistochemistry. In the literature, we found a few articles about IFN-γ expression in epithelial cells, which may be absorbed by the surrounding immune cells. Xu et al. found that INF-γ is upregulated in resected gastric cancer tissue. Additionally, the authors claim that in GC cell culture, IFN-γ stimulates the nuclear factor NF-kb signaling pathway and enhances proliferation, migration and invasion, and inhibition of IFN-γ could be essential for the treatment of GC. After stimulation with IFN-γ but not TNF-γ, PD-L1 is expressed in a dose-dependent manner in GC cell culture, the supernatant and the cell membrane.

Moreover, the serum of patients with advanced GC contains a higher amount of free PD-L1 than healthy controls [34]. Contradictorily, in clinical practice, patients who do not respond to CTLA-4 immunotherapy are those with genomic defects of IFN-γ pathway genes [35]. Mimura et al. found that in vitro IFN-γ stimulates PD-L1 expression through the JAK signal transducer and activator of the transcription pathway and impairs the cytotoxicity of tumor-antigen-specific CTL against tumor cells [36]. We found INF-γ in 31% of cases in cancer cells in tumor and metastasis. This expression is correlated with the presence and the number of CTLA-4-positive cells in the lymph node. Additionally, IFN-γ-positive GCs had IFN-γ-positive immune cells more frequently in tumor infiltration areas, invasive margin areas and lymph nodes. There was no association of IFN-γ positivity in tumor cells with the PD-L1 tumor status.

The response signature involves the expression of inflammatory genes associated with the adaptive immune response, including IFN-γ as a major one. According to some genetic studies, immune suppressive elements in the TME, combined with an inflamed signature, may more accurately predict the outcome of immunotherapy [37]. Some experimental studies show the effect of IFN-γ on gastric mucosal cells that undergo metaplasia or death after exposure [30]. Imai et al. found that IFN-γ stimulates PD-L1 expression in tumor cells in vitro and is increased in the serum of patients with stage IV GC [34].

## 5. Conclusions

PD-L1 expression in tumor cells correlates with the intestinal type, number of CD8^+^T cells and macrophages in the TME of GC, and IFN-γ expression in tumor cells correlates to the presence of suppressive CTLA-4^+^/IFNγ^+^ immune cells in the TME and the lymph node. Considering all improvements in GC therapy, we are still pursuing more effective therapeutic targets that will unleash T-cell reactivity in tumors. Therefore, new insight into cellular and molecular tumor interactions will pave the way for new treatment modalities that will build on the biological characteristics of the TME.

## Figures and Tables

**Table 1:** Demographic and clinicopathological data of patients with GC.

Variable		N (%)
Gender	male	66 (68)
	female	34 (32)
Histologic type	intestinal	61 (63)
	diffuse	34 (35)
	mixed	2 (2)
Feature	polypoid	16 (16.5)
	fungoid	40 (41.2)
	ulcerative	17 (17.5)
	flat/infiltrative	24 (24.7)
Stage	IA	10 (10.3)
	IB	4 (4.1)
	IIA	14 (14.4)
	IIB	17 (17.5)
	IIIA	24 (24.7)
	IIIB	25 (25.8)
	IVB	3 (3.1)
IFN-γ in tumor cells	negative	60 (61.9)
	low	17 (17.5)
	moderate	9 (9.3)
	strong	11 (11.3)
IFN-γ in metastasis	negative	63 (64.9)
	low	14 (14.4)
	moderate	10 (10.3)
	strong	10 (10.3)
CTLA-4^+^ in TI	M Q1-Q3; min–max	0 (0–3; 0–35)
CTLA4^+^^+^/IFNγ^+^		1 (1–3; 0–55)
CTLA-4^+^ in IM		0 (0–1; 0–26)
CTLA4^+^^+/^IFNγ^+^		0 (0–2; 0–74)
CTLA-4^+^ in LN		0 (0–1; 0–11)
CTLA4^+^^+^/IFNγ^+^		0 (0–1; 0–20)
PD-L1	negative	66 (68)
	low	18 (18.6)
	moderate	10 (10.3)
	strong	3 (3.1)
CD8+ T-cell		22 (11–38; 3–186)
macrophages	negative	48 (49.5)
	low	23 (23.7)
	moderate	13 (13.4)
	strong	13 (13.4)

TI—tumor infiltration area, IM—invasive margin area, LN—lymph node.

**Table 2:** Correlation of PD-L1 status of GC with clinical and pathological parameters.

	PD-L1 Status of Tumor		*p*	OR (95 CI)	*p* Log-Rank Test
	Negative N = 66 (68.1%)	Positive N = 31 (31.9%)			
Age (years)	73:64–79; 46–85	(76:67–82; 51–87)	0.181†		
Gender					
m	47 (71)	19 (61)	0.457 *		
f	19 (29)	12 (39)			
Histologic type					
intestinal	38 (58)	25 (81)	0.046 *	(3.1; 1.1–8.5)	0.030
diffuse	28 (42)	6 (19)			
feature			0.239 *		
polypoid	9 (13.6)	7 (22.6)			
fungating	25 (37.9)	15 (48.4)			
ulcerated	12 (18.2)	5 (16.1)			
flat/infiltrating	20 (30.3)	4 (12.9)			
Stage			0.057 *		
IA, IB	11 (17)	3 (10)			
IIA, IIB	16 (24)	15 (48)			
IIIA, IIIB, IVB	39 (59)	13 (42)			
CD8^+^ T cells	15:9–27; 3–118	(39:27–56; 3–186)	<0.001 ^†^	(9.8; 3.6–26)	0.001
≥28.5	51 (77)	8 (26)			
<28.5	15 (23)	23 (74)			
IFN-γ in tumor cells			0.181 *		
negative	40 (60.6)	20 (64.5)			
low	9 (13.6)	8 (25)			
moderate	7 (10.6)	2 (6.5)			
strong	10 (15.2)	1 (3.2)			
INF-γ in metastasis			0.122 *		
negative	60 (60.6)	23 (74.2)			
low	8 (12.1)	6 (19.4)			
moderate	9 (13.6)	1 (3.2)			
strong	9 (13.6)	1 (3.2)			
Macrophages			<0.001 *	(13.5; 4.2–43)	
negative	44 (67)	4 (13)		-	
low, moderate, strong	22 (33)	27 (87)			
CTLA-4 in TI *	(0:0–4; 0–35)	(0:0–2; 0–20)	0.159 ^†^		
CTLA-4			0.188 *		
negative	36 (54)	22 (71)			
positive	30 (46)	9 (29)			
CTLA-4 in IM *	0:0–1; 0–26	(0:0–0; 0–7)	0.197 ^†^		
CTLA-4					
negative	45 (68)	25(81)	0.301 *		
positive	21 (32)	6(19)			
CTLA-4 lymph node	0:0–1; 0–11	(0:0–0; 0–4)	0.292 ^†^		
CTLA-4					
negative	40 (61)	15 (48)	0.361 *		
positive	26 (39)	16 (52)			

* chi-square test. ^†^ Mann–Whitney U test.

**Table 3:** Multivariate analysis of variables associated with PD-L1 status of GC.

Variable	OR (95%CI)	*p*
Histologic type	0.429 (0.12–1.5)	0.182
intestinal		
diffuse		
Macrophage infiltration	9.8(2.7–35)	<0.001
no		
mild to strong		
CD8^+^ T cells	7 (2.3–21)	<0.001
≥28.5		
>28.5		
Stage	0.86 (0.38–2)	0.731
IA, IB		
IIA, IIB		
IIIA, IIIB, IVB		

**Table 4:** Correlation of IFN-γ in tumors with CTLA-4 lymphocytes in gastric cancer.

Lymph Node	Gastric Cancer		*p*	OR (95%CI)	*p* Log-Rank Test
	IFN-γ-Negative (N = 60)	IFN-γ-Positive (N = 37)			
CTLA-4^+^ lymphocytes	0 (0–1; 0–10)	1 (0–2; 0–11)	0.027		
CTLA-4^+^ lymphocytes			0.059	2.4 (1.1–5.6)	0.037
negative	39 (65)	16 (43)			
positive	21 (35)	21 (57)			

**Table 5:** The correlation of the CTLA-4/IFN-γ lymphocytes with the PD-L1 status of GC.

Area CTLA-4/IFN-γ Lymphocytes	PD-L1 Tumor Status		*p*
	Negative	Positive	
TI			0.638
−/−	24 (36.4)	15 (48.4)	
+/−	16 (24.2)	5 (16.1)	
−/+	12 (18)	6 (19.4)	
+/+	14 (21.2)	5 (16.1)	
			0.612
TM			
−/−	28 (42.4)	17 (54.8)	
+/−	12 (18.)	3 (9.7)	
−/+	18 (27.3)	8 (25.8)	
+/+	8 (12.1)	3 (9.7)	
LN			0.069
−/−	28 (42.4)	10 (32.3)	
+/−	10 (15.2)	12 (38.7)	
−/+	12 (18.2)	5 (16.1)	
+/+	16 (24.2)	4 (12.9)	

TI—tumor infiltration area, IM—invasive margin area LN—lymph node.

**Table 6:** Correlation of the CTLA4/IFN-γ lymphocytes with IFN-γ expression in GC.

Area	CTLA4/IFN-γ Lymphocytes	IFN-γ Tumor Cells		*p*
		No (60)	Yes (37)	
TI				<0.001
	−/−	39 (65)	0	
	+/−	21 (35)	0	
	−/+	0	18 (49)	
	+/+	0	19(51)	
TM				<0.001
	−/−	45 (75)	0	
	+/−	15 (25)	0	
	−/+	0	26 (70)	
	+/+	0	11 (30)	
LN				<0.001
	−/−	37 (61.7)	1 (2.7)	
	−/+	19 (31.7)	3 (8.1)	
	+/−	2 (3.3)	15 (40.5)	
	+/+	2 (3.3)	18 (28.6)	

TI—tumor infiltration area, TM—tumor invasive margin, LN—lymph node.

**Table 7:** Mean general survival and median overall survival according to analyzed parameters in patients with GC.

Variable	General Survival (95%CI)	Median Overall Survival (95%CI)	LR; *p*
CTLA4^+^ cells, TA			0.569; 0.451
negative	29 (4) (22–37)	17.5 (2.4; 13–22)	
positive	35 (5.5; 24–46)	14.5 (9; 1–31)	
CTLA4^+^ cells, TI			0.001; 0.976
negative	31.6 (3.7) (24–39)	17.5 (4) (9.7–25)	
positive	32 (6.5) (19.5–45)	11.3 (6.8) (1–25)	
CTLA4^+^ cell, LN			2.2; 0.137
negative	26 (3.7 (19–33))	14.5 (3.8) (7.1–22)	
positive	37 (5) (27–47)	22(6) (9.6–34)	
Gender			0.818; 0.366
male	29 (3.7) (22–37)	16 (4) (7.6–25)	
female	35 (6) (24–47)	17.5 (7.5) (3–32)	
Histologic type			0.166; 0.684
intestinal/mixed	33 (4) (25–41)	16 (3) (10–22)	
diffuse	27 (4) (19–36)	19 (7) (6–32)	
Macrophages			2.3; 0.503
polypoid	21 (5.6) (10–32)	11 (2) (7–15)	
fungate	35 (5) (25–45)	22 (6) (9–35)	
ulcerative	23 (5) (13–34)	15 (7) (1.6–29)	
flat/infiltrating	36 (7) (22–49)	19 (8) (3–35)	
Disease stage			10.9; 0.004
IA, IB	55.8 (7) (42–69)		
IIA, IIB	28.3 (6) (17–39)	15.7 (9) (1–33)	
IIIA, IIIB, IVB	25.6 (4) (18–33)	13 (2) (9–18)	
CD8 + T cells (mean)			0.967; 0.326
≤28.5	29 (4) (21–37)	14.5 (3) (8–20)	
>28.5	36 (5) (25.5–46)	24 (11) (2–46)	
IFN-γ in tumor cells			3.8; 0.286
no	32.4 (4) (24–40)	18 (4) (10–26)	
low	24 (6) (12–36)	13.5 (6) (2–25)	
moderate	17 (5) (6.6–28)	14.5 (11) (0–36)	
strong	47 (12) (24–70)		
IFN-γ in metastasis			2.5; 0.476
no	32.5 (4) (25–40)	22 (4) (14–29)	
low	24 (7) (9.6–38)	11 (5) (1.5–20)	
moderate	19.9 (7) (6–33)	7 (7) (0–21)	
strong	43 (12) (19–67)	11	
Macrophages			0.667; 0.881
no	32 (4.6) (23–41)	18.6 (5) (8–29)	
sparse	33 (6) (20–45)	16 (3.6) (8.7–23)	
moderate	26 (8) (11–41)	16 (10) (0–36)	
strong	25 (6) (13–38)	11 (14) (0–38)	
Macrophages			0.09; 0.41
no	32 (4.6) (23–41)	18.6 (5) (8–29)	
sparse to strong	31 (4) (22–39)	16 (3) (9–22)	
PD-L1			0.599; 0.439
negative	29 (4) (22–37)	14.5 (4) (7–22)	
positive	36 (6) (25–47)	30 (9) (12–48)	

TA—tumor-infiltrating area, TI—tumor invasive margin, LN—lymph node.

## Data Availability

Data are available upon request.
